# Multidimensional Connectomics and Treatment-Resistant Schizophrenia: Linking Phenotypic Circuits to Targeted Therapeutics

**DOI:** 10.3389/fpsyt.2018.00537

**Published:** 2018-10-30

**Authors:** Mary-Anne B. MacKay, John W. Paylor, James T. F. Wong, Ian R. Winship, Glen B. Baker, Serdar M. Dursun

**Affiliations:** Neurochemical Research Unit and Bebensee Schizophrenia Research Unit, Department of Psychiatry, University of Alberta, Edmonton, AB, Canada

**Keywords:** schizophrenia, treatment-resistant, connectomics, dysconnectivity, gamma band oscillations, NMDA receptors, GABA interneurons

## Abstract

Schizophrenia is a very complex syndrome that involves widespread brain multi-dysconnectivity. Neural circuits within specific brain regions and their links to corresponding regions are abnormal in the illness. Theoretical models of dysconnectivity and the investigation of connectomics and brain network organization have been examined in schizophrenia since the early nineteenth century. In more recent years, advancements have been achieved with the development of neuroimaging tools that have provided further clues to the structural and functional organization of the brain and global neural networks in the illness. Neural circuitry that extends across prefrontal, temporal and parietal areas of the cortex as well as limbic and other subcortical brain regions is disrupted in schizophrenia. As a result, many patients have a poor response to antipsychotic treatment and treatment failure is common. Treatment resistance that is specific to positive, negative, and cognitive domains of the illness may be related to distinct circuit phenotypes unique to treatment-refractory disease. Currently, there are no customized neural circuit-specific and targeted therapies that address this neural dysconnectivity. Investigation of targeted therapeutics that addresses particular areas of substantial regional dysconnectivity is an intriguing approach to precision medicine in schizophrenia. This review examines current findings of system and circuit-level brain dysconnectivity in treatment-resistant schizophrenia based on neuroimaging studies. Within a connectome context, on-off circuit connectivity synonymous with excitatory and inhibitory neuronal pathways is discussed. Mechanistic cellular, neurochemical and molecular studies are included with specific emphasis given to cell pathology and synaptic communication in glutamatergic and GABAergic systems. In this review we attempt to deconstruct how augmenting treatments may be applied within a circuit context to improve circuit integration and treatment response. Clinical studies that have used a variety of glutamate receptor and GABA interneuron modulators, nitric oxide-based therapies and a variety of other strategies as augmenting treatments with antipsychotic drugs are included. This review supports the idea that the methodical mapping of system-level networks to both on (excitatory) and off (inhibitory) cellular circuits specific to treatment-resistant disease may be a logical and productive approach in directing future research toward the advancement of targeted pharmacotherapeutics in schizophrenia.

## Introduction

Treatment-resistant schizophrenia (TRS) remains one of the greatest therapeutic challenges in psychiatry. Schizophrenia is a complex neurodevelopmental syndrome; with disease processes occurring *in utero* that may disrupt the formation of critical neural circuits and result in widespread brain dysconnectivity. Hints of altered neural circuitry, for example delays in gross and fine motor skill development, often evolve during childhood and may precede the first subtle signs of psychosis during late adolescence in those who will develop the illness (1–4). Adolescents with disrupted neural circuit development and circuit dysconnectivity related to the progression of the disease often begin to exhibit sub-threshold psychotic symptoms during developmental periods associated with increasing gray matter (GM) volume and refinement of cortical circuits including synaptic pruning, reinforcement, and neuronal synchronization (5–8). The gradual alterations in brain connectivity and subsequent symptoms can persist for years before psychosis emerges and diagnosis and antipsychotic medications are initiated. In most cases, individuals with schizophrenia progress with an illness that is characterized by periods of exacerbation and remission of psychosis. Recovery is dependent on compliance with and response to optimized antipsychotic medication, the development of a strong therapeutic alliance to treatment team members, and intensive social and vocational support (9). Even with the best antipsychotic treatments that are available today and access to full functional supports, a sub-population of patients with schizophrenia will never attain an optimal response to treatment and remain very ill. These are the patients who have treatment-refractory illness or in the case of non-response to clozapine, ultra-resistant disease (10, 11).

Identifying treatments that will benefit patients with TRS remains a significant challenge. Our understanding of personalized treatment response and resistance to medication is limited by an inability to accurately pinpoint the individual genetic, cellular and neural circuit drivers of psychoses. Investigations of neuronal ensembles and cortical networks at the micro-scale level are not possible using the clinical diagnostic and macro-scale imaging tools that are currently available. Moreover, inconsistent clinical definitions of positive, negative or cognitive symptom-specific differences in TRS lead to ambiguous treatment guideline recommendations and a wide variation in clinical approaches to treat TRS in practice. Different phenotypes of psychoses may respond to different targeted treatments that are cellular or neural circuit-specific, but at present we do not have the ability to identify the appropriate targeted therapies for different TRS phenotypes.

The Treatment Response and Resistance in Psychosis (TRRIP) working group recently addressed these challenges (12). Members are researchers and clinicians who have expertise in TRS and attended specific TRRIP working group meetings at international schizophrenia and neuropsychopharmacology research conferences to establish criteria to standardize the definition of treatment resistance in schizophrenia. In addition to capturing a core definition of treatment resistance that can be included and shared across all clinical treatment guidelines worldwide, recommendations were also made on the importance of identification of all clinical sub-specifiers or symptom phenotypes common to TRS (12). The standardization of clinical criteria of TRS has been an important advancement and will benefit future TRS research and clinical translation.

Treatment resistance has been most characterized in schizophrenia by how responsive the positive symptom domain is to antipsychotic medications. It is estimated that 70–80% of patients with schizophrenia have a phenotype of psychosis that is responsive to dopamine-blocking treatment (13). However, in over 100 years of treatment history and despite the improvements made to the functional selectivity and potency of antipsychotic medications, 60% of patients continue to fail to achieve symptom improvement after several weeks on drug therapy (14).

Many treatment-refractory patients present with a psychosis that is positive symptom domain responsive, but have symptoms that are non-responsive within the negative or cognitive symptom subdomains and associated circuits. It is now recommended that patients with symptom profiles that do not respond to antipsychotic medication and are considered treatment resistant be identified as: TRS-positive symptom domain-, TRS-negative symptom domain-, and TRS-cognitive symptom domain-specific. For those patients with combined treatment resistance in more than one domain (multidimensional resistance), identifying all of those specific symptom domains will provide further clarification (12).

Traditionally, for those patients who are unable to obtain adequate positive symptom control or sustain a response with at least 2 dopamine receptor-2 (D_2_)-blocking agents at therapeutic doses for at least 6 weeks, clozapine is the recommended drug of choice. An estimated 30–60% of these patients will respond to clozapine and have what can be described as a clozapine-responsive psychosis (10, 15, 16). Patients who do not have an optimal response to clozapine and continue to experience prominent positive symptoms have clozapine-resistant psychosis or an ultra-resistant psychotic disease (11). Currently, there are no therapies that address this most severe form of neural-dysconnectivity in schizophrenia.

In this review, we examine TRS from a circuit-based perspective. We start by highlighting the historical development of connectome science in schizophrenia, identifying those early pioneers in psychiatry who originally recognized the disease as an illness of widespread disconnectivity and their valuable contribution to the evolution of network science today. We then examine neuroimaging studies that support both systemic and circuit-level brain dysconnectivity specific to treatment resistance and attempt to explain underlying circuit biology and brain topology that may be unique to this most severe form of the illness. Within a connectome context, attempts to map on-off circuit connectivity synonymous with excitatory and inhibitory neuronal pathways are discussed. Functional correlates of dysconnectivity in schizophrenia are also considered with a focus on cortical network oscillations, giving particular emphasis to the role of gamma band oscillations (GBOs) and their ability to integrate information across large populations of neurons in the illness. Mechanistic models describing underlying neural circuitry and the complex relationship involved in the synchronized firing between excitatory pyramidal cells and inhibitory gamma-aminobutyric acid (GABA)-ergic interneurons are also reviewed to help visualize and understand the inter-relationship between neuronal ensembles within the brain and the complex mechanisms behind their dysfunctional communication in schizophrenia. Finally, we deconstruct how augmenting pharmacological treatments, such as glutamate *N*-methyl-D-aspartate (NMDA) receptor and GABA interneuron modulators as well as nitric oxide (NO)-based treatments may be applied within a circuit context to improve circuit integration and treatment response in TRS. Updates on neurosurgical and neuromodulation targets under investigation in TRS are also included and provide an overview of beneficial circuit-based targets that may improve treatment resistant symptoms in those patients that remain refractory to pharmacological approaches.

This review supports the idea that the mapping of cellular and system-level networks to both on (excitatory) and off (inhibitory) circuit phenotypes specific to treatment-resistant disease may be a productive strategy in expanding future research toward customized neural circuit-specific pharmacotherapeutics and directed neuromodulation treatments in schizophrenia. Targeted therapeutics that can improve particular areas of regional functional dysconnectivity that are found to be substantially affected in TRS is an intriguing approach to precision medicine in schizophrenia.

## History of connectomics in schizophrenia-the early connectionists

Theoretical models of disconnectivity and the investigation of connectomics and brain network organization have been examined in schizophrenia since the early nineteenth century. Historically, there have been a number of influential figures who have made major contributions to the development of modern day network-based science known as connectomics. One of the very first connectionist pioneers in psychiatry was Wilhelm Griesinger (1817–1868), a German neurologist and psychiatrist who initially proposed that mental illnesses are brain disorders with pathological and neuroanatomical origins similar to neurological disorders (17). From his teachings, his student Theodor Hermann Meynert (1833–1892), a German-Austrian neuropathologist, anatomist and psychiatrist, made further contributions to this biological model of mental illness (18). His work was based primarily on neuroanatomical and histological studies where he worked to characterize various afferent and efferent white matter (WM) fiber tracts of the cerebral cortex. Meynert believed that association fibers connecting regional areas of the brain are the most disrupted in psychiatric diseases, which has been consistently demonstrated by several structural and functional magnetic resonance imaging (MRI) studies of schizophrenia in recent times (18–21).

Meynert's student Carl Wernicke (1848–1905) further developed the disconnectivity theory of schizophrenia. Although he was best known for his theories regarding the neural circuits involved in higher cognitive functions and the neuropathology of aphasia, he also studied the neuroanatomical and functional aspects of schizophrenia. In his textbook *Grundriss der Psychiatrie* (Outlines of Psychiatry 1900) which was written based on detailed reviews of his clinical cases, he outlined his hypothesis that there is a deficiency in association fiber connectivity in schizophrenia that contributes to an over-activation of cortical sensory regions that can then lead to the development of psychosis (22).

One of the most well-known clinicians in the history of psychiatry and recognized as the founder of modern psychiatry was Emil Kraepelin (1856–1926), a German psychiatrist who conceptualized schizophrenia as a disorder with both neurodevelopmental and biological origins. Kraepelin was the first to develop a classification system of psychiatric disorders and divided endogenous psychoses into two distinct forms based on disease course and outcome. He described the psychosis involved in schizophrenia as a dementia praecox, a term that combined the cognitive symptoms (dementia) of the illness with an early development of the disorder (praecox) vs. the episodic nature of manic depressive (affective) psychosis (23).

It was the Swiss psychiatrist Eugen Bleuler (1857–1939) who then coined the term schizophrenia (from the Greek verb *schizein* meaning split and *phren* meaning soul, spirit or mind) to highlight the fragmented thinking or thought disorder that is common to the functional disconnectivity of the illness. Bleuler replaced the term dementia praecox to clearly distinguish schizophrenia from a degenerative illness with a poor outcome. He recognized that progressive cognitive deterioration (characteristic of dementia) was not common in schizophrenia and the onset of symptoms does not always occur early in life (24). For a detailed overview see Collin et al. (19).

## Modern-day connectionists

With the advancement of neuroimaging techniques, such as positron emission tomography (PET) and MRI that are able to detail both anatomical and functional connectivity, the disconnection hypothesis of schizophrenia has been refined further. The modern-day disconnectivity hypothesis of schizophrenia initially emphasized the link between the signs and symptoms of schizophrenia and the dysfunctional integration between different cortical areas of the brain, directly related to the underlying abnormalities in neurons and synaptic functioning (25). Abnormal modulation of NMDA receptor function and impaired control of synaptic plasticity is thought to be the underlying key to dysfunction and directly contributes to an extended pattern of “dysconnection” of the structural and functional integration of the brain (26–30). Today, network scientists integrate the mathematical analysis of graph theory as a framework for studying and tracing these macro-scale brain networks through non-invasive neuroimaging and MRI methods (31–33). Through these methods they are able to create a “connectome,” the neuronal map of the brain's anatomical and functional connectivity architecture, and elucidate the complex organization of the neuronal elements that underlies brain function (31–34).

## The science of connectomics

The scientific study of connectomics involves mapping out the detailed connectivity of brain regions to characterize the architectural networks of the human brain. Connectomics is therefore a powerful tool to visualize the structural and functional dysconnections associated with schizophrenia. The human connectome provides a detailed map of brain-wide circuit connectivity and allows inference into how brain function may be affected by disruption of the structural organizational network (31, 34). At the micro-scale, the physical wiring of single neurons and their synaptic connections to other neurons through dendritic and axonal connections comprise local network circuits. At the meso-scale (local populations of 80–100 neurons that span all cortical layers), connectivity is at the level of functionally specialized subnetworks within single cortical columns that are selectively connected within and between neighboring cortical columns and constitute a major functional element for cortical information processing. At the macro-scale, inter-regional connectivity of cerebral lobes via WM interhemispheric tracts is responsible for the integration and relay of information between various parts of the brain (34).

**Figure 1:**
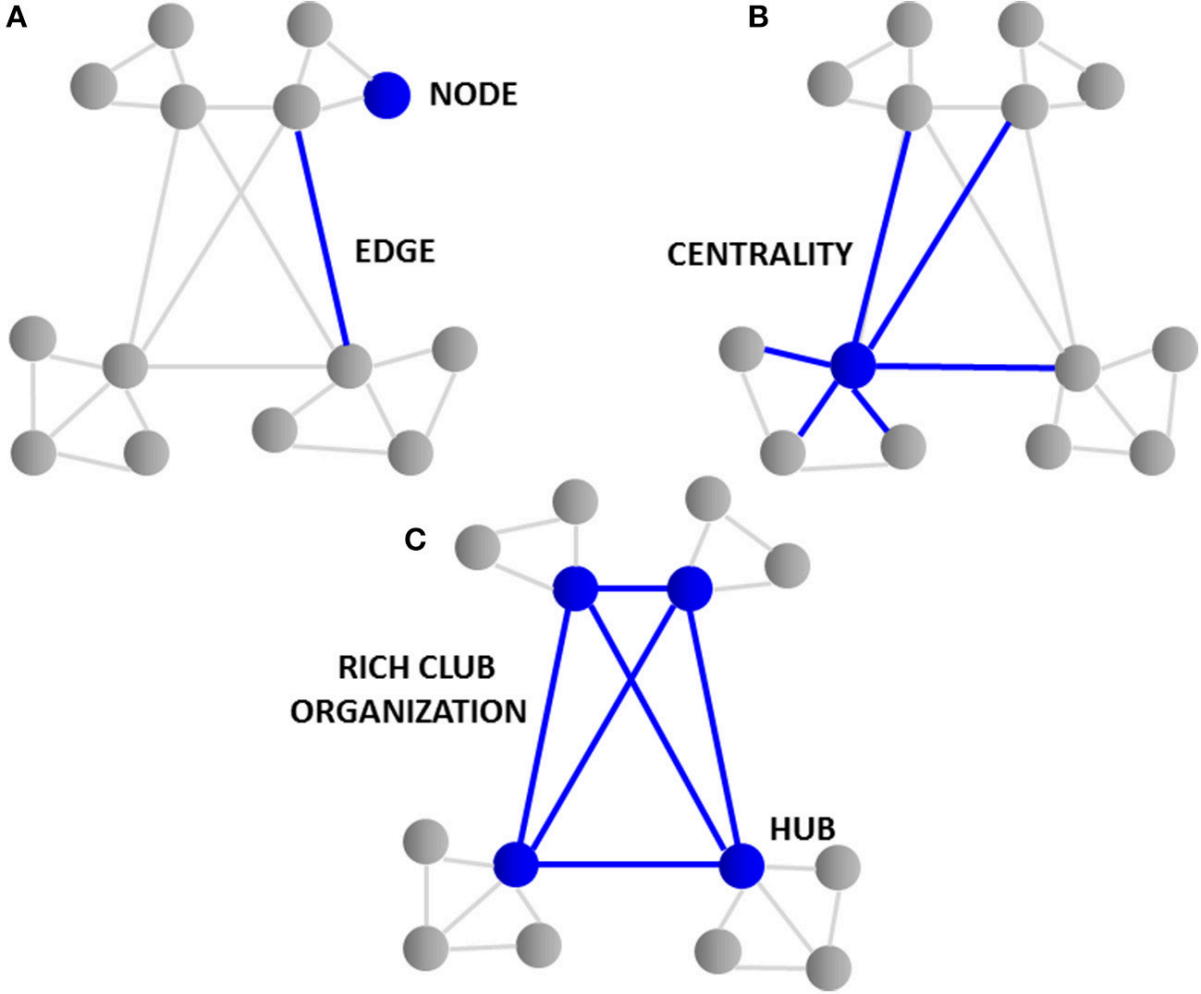
Topological graph features of the connectome. **(A)** The graph consists of “nodes” that represent single neurons or brain regions and are linked by “edges” illustrating their connection to each other via axonal projections. **(B)** The “degree” or number of edges and how close they are to each other “centrality” represents the interconnectivity of nodes. **(C)** Nodes having a high degree of edges and high centrality are knowns as “hubs.” Brain hubs “rich” in connectivity to each other and found centrally form the “rich club organization.” The rich club hubs found in cortical and frontal lobe regions of the brain are affected in schizophrenia.

Connectomics heavily utilizes graph theory, a specialized discipline of mathematics concerned with the study of graphs or models that represent relations between objects. Large collections of algorithms are used to calculate topological characteristics of both structural and functional brain imaging connectivity data that can be represented in the form of a graph. The graph consists of nodes that represent single neurons or brain regions that are defined by connection endpoints of two line segments that are then linked by edges that illustrate their direct connection to each other via axonal projections, WM pathways or functional coupling between inter-regional brain areas (31). The closeness of neuronal and brain region nodes represents a higher probability of being connected, as long axonal projections are functionally expensive in terms of wiring costs. The tendency of nodes to cluster and form shorter communication paths allows for more efficient integration between spatially disconnected node pairs. The degree or number of edges each node possesses and how close they are to each other (centrality) represents the interconnectivity of a node to other nodes within the entire brain network. Nodes that have a high degree of edges and possess high centrality are known as hubs (35–37). In turn, brain hubs that are “rich” in connectivity and more densely interconnected to each other in comparison to what their high degree alone would predict form a central “rich club organization” essential for the integration of global information and brain communication (37) as illustrated in Figure 1. Disruption to central “rich club” hubs of the human connectome has been associated with several brain disorders (38). Notably, hub lesions that are highly concentrated within cortical hubs of the frontal and temporal lobes are found to be specifically affected in schizophrenia (38).

## The connectomics of schizophrenia

Neuroimaging studies show impaired structural and functional connectivity in individuals diagnosed with schizophrenia (39–41). The dysconnection between different brain regions of GM and the WM circuits that connect them are consistent with reduced functional connectivity revealed in both resting state and task-based functional (fMRI) studies (32, 41). Recent advances in the use of MRI and in particular diffusion-weighted imaging (DTI) have brought insight into the extent of structural WM dysconnectivity and alterations in the macro-scale neuronal wiring in schizophrenia. Most studies have investigated fractional anisotropy (FA), a neuroimaging marker that indexes the constraint of the direction of water diffusion in WM and can be a measure of an abnormality in the integrity of myelin microstructure or axonal integrity or differences in the orientation of how axonal fibers are organized. White matter in the frontal and temporal lobes have been the most frequently reported with reduced FA integrity in DTI studies of those with schizophrenia (39–41). Meta-analyses of voxel-based DTI studies in schizophrenia have found significant decreases in two main brain regions, the left frontal deep WM and left temporal deep WM (42), with overlapping GM and WM structural abnormalities (43). A more recent meta-analysis that included 29 independent international studies found global WM microstructural disruptions throughout the entire brain (44).

Consistent with these findings, an additional imaging study found significant decreases in WM FA to not only involve the fronto-temporal regions, but also to be widespread throughout each lobe of the brain, including the cerebellum. Major fiber bundles that connect the cortical lobes including the corpus callosum, cingulum and thalamic radiations exhibited the most severe pathology. More than 50% of the cortico-cortical and cortico-subcortical WM fibers that provide the connections between those hub regions that contribute to the “rich club” in the brain were affected (45) and network hubs located in association cortex particularly affected (20, 21). These significant structural disturbances may be responsible for the widespread disruption of cortical information processing and integration of information across multiple regions of the brain in schizophrenia.

Functional MRI studies have also suggested abnormalities in the connectivity of brain networks in schizophrenia and relate to the structural disturbances that interconnect them. While reduced functional connectivity is a replicated finding among many studies (32, 41, 46, 47), there have also been reports of increase in functional connectivity in the illness (48, 49). The discrepancy may simply be related to non-uniform changes in brain connectivity, such as hyper-synchrony of neuronal ensembles vs. dysregulated networks, fMRI preprocessing errors, or abnormalities in neuronal wiring and oscillatory firing and compensatory hyper-connectivity of important hubs within the association cortex as a consequence of the illness (50).

## The connectomics of treatment resistance

Widespread dysfunction throughout the entire neural network that involves both cortical and subcortical regions is pronounced in TRS and may have an underlying circuit biology that is unique to this most severe form of the illness. Anatomical regions and neural circuits that have been examined comparing those individuals with treatment resistant vs. treatment responsive disease have uncovered more severe pathological findings in all cortical tissues that have been measured. A number of imaging studies using a variety of structural and fMRI methods have examined TRS to elucidate the difference between the phenotypic subtypes of responsive and non-responsive illness. For detailed reviews see Mouchlianitis et al. (51) and Nakajima et al. (52).

The loss of neuronal elements that underlie the symptoms of both TRS and ultra-resistant schizophrenia (clozapine-resistant psychosis) may be more substantial than what is found in those patient phenotypes who have responded to antipsychotic treatment (51, 52). Volumetric, DTI and fMRI studies that have examined intra-regional brain morphology (53–56) inter-regional WM circuit integrity (43, 57–59), and functional connectivity (60–63) specific to TRS have consistently identified a disruption to frontal and temporal lobe regions and the major fiber bundles that connect them.

Studies that have specifically compared patients with treatment responsive schizophrenia vs. TRS have reported greater global volumetric reductions of GM in treatment resistant and ultra-resistant patients. There have been consistent reports of reduced GM volumes predominantly within the dorsolateral prefrontal cortex (DLPFC) (53–56), as well as posterior cortical regions, such as the temporal cortex (53–56), parietal cortex (53, 56) and also within the occipital cortex (53, 55, 56) in TRS. Abnormalities in all regions of the corpus callosum as well as commissural and association long axonal fiber pathways connecting prefrontal, temporal, parietal and occipital regions have also been found, with reduced axonal integrity and more severe structural damage in both chronic illness and treatment-resistant populations (43, 57–59, 64). This evidence seems to suggest that on the spectrum of cellular and circuit disruption characteristic of schizophrenia in general, TRS may involve a more severe type of multi-dysconnectivity of brain networks that spans across almost every region of the brain.

The reduction in cortical GM and WM volumes and distinct WM tract disturbances in TRS may be a consequence of disrupted macro-scale neural architecture and network dysconnectivity that originate within distinct micro-scale neuronal ensembles. Morphometric studies that have been investigated in schizophrenia suggest that cortical volume loss is not related to the reduction of the number of neurons in the cortex, but to architectural neuronal disorganization, reduction in neuronal size, and diminished neuropil (axons, dendrites, and synaptic terminals) (65, 66). The etiology behind the loss of dendritic spines and dendritic length of cortical pyramidal neurons is not entirely clear but may originate from hypofunctioning NMDA glutamate receptors on pyramidal cells and interneurons (67–69). From a circuit perspective, hypofunction of NMDA receptors on GABAergic inhibitory interneurons disinhibits associated pyramidal neurons in the circuit and causes a potentially pathological glutamatergic excitatory effect (70, 71).

Hyperglutamatergia may be a distinct feature of TRS and be differentiated from treatment-responsive disease since greater abnormalities in glutamate function have been found in those patients with TRS while maintaining a relatively normal and intact dopamine function. Neuroimaging measures using fluorine-18-L-dihydroxyphenylalanine (^18^F-DOPA) as a PET radiotracer found a higher level of striatal dopamine synthesis capacity in patients with schizophrenia who responded to treatment vs. those patients with TRS who had equivalent striatal dopamine levels found in healthy controls (72). The same group later utilized proton magnetic resonance spectroscopy (^1^H-MRS) imaging in TRS to examine glutamate changes that may be specific to antipsychotic treatment-resistance (73). This was the first group to report high glutamate and glutamine levels in the anterior cingulate cortex (ACC) in TRS as compared to those with schizophrenia in remission, and another group has since replicated this finding (74).

Increased concentrations of glutamate found in the ACC that are specific to TRS are consistent with both the glutamate hyperfunction and the NMDA receptor hypofunction hypotheses of schizophrenia. Normally, glutamate is responsible for regulating inhibitory tone in the brain by binding to NMDA receptors on GABAergic interneurons. The structural mechanism that may cause NMDA receptor hypofunction in TRS can lead to disinhibition of pyramidal neurons and excitatory pathways by the understimulation of inhibitory GABA interneurons (75). The downstream effect can then cause an increase in glutamate release from presynaptic pyramidal neurons and binding to α-amino-3-hydroxy-5-methylisoxazole-4-propionic acid (AMPA) and kainate receptors and may be a compensatory effect of the NMDA blockade (75–78). The hyperglutamatergic state can initiate calcium influx and cellular toxicity which, over time, can be detrimental to neuronal networks (79). In treatment-resistant disease, excitatory inputs from pyramidal neurons within the ACC circuit could also be disinhibited, leading to increased glutamate efflux and generating symptoms that fail to respond to D_2_-blocking medications. Glutamate-mediated excitotoxicity may be responsible for the widespread brain abnormalities and severity of symptoms that are found in TRS.

Disturbances in the functional activity of neural circuits have consistently been reported in TRS. Functional MRI studies that have examined changes in neurophysiological measures also may indicate disordered firing and pathological oscillatory activity that may be more pronounced in TRS (63). Persistent auditory hallucinations are a core feature of psychosis. Poor control of this symptom within the positive symptom domain that persists despite adequate trials of antipsychotic medications is often the clearest and most common indicator of severe treatment resistance. Patients with specific TRS-positive symptom domain phenotypes and experiencing auditory verbal hallucinations (AVH) have been investigated in fMRI studies (60–63).

Functional MRI using magnetically labeled blood water protons as an endogenous tracer (arterial spin labeling) to measure tissue perfusion found increased cerebral blood flow in the left superior temporal gyrus, right supramarginal gyrus, and temporal polar cortex in patients with treatment-resistant AVH (63). Functional resting-state MRI studies that investigated connectivity alterations in the default network in patients with chronic non-responsive AVH and treated patients without AVH found that treatment-resistant patients had increased functional connectivity between the dorsomedial prefrontal cortex and other frontotemporal regions, but reduced connectivity between the ventromedial prefrontal cortex and areas of the cingulate cortex (60). Reduced functional connectivity between the left temporo-parietal junction (TPJ) and right Broca's area and ACC and temporo-cingulate pathways have also been implicated in patients with persistent AVH (61, 62). All functional alterations found were greater in those patients with persistent treatment-resistant symptoms, indicating there may be fundamental differences within these brain network properties that are also specific to TRS.

Network-based statistics can be applied to fMRI data to investigate brain networks and to better delineate the differences in the connectome unique to TRS. Although there have been a number of network-based studies in schizophrenia (31, 32, 45–47), Ganella et al. were the first to measure the connectivity and global and local efficiency of whole-brain functional networks from resting state fMRI data in individuals with TRS compared to healthy controls (80). Whole-brain connectivity analysis in this study showed reductions in functional connectivity between all of the brain lobes, with the majority of reduced connections between fronto-temporal, fronto-occipital, temporo-occipital and temporo-temporal subregions. The majority of reduced functional connections in TRS were found in the temporal lobe (between Heschl's gyrus and the frontal lobe), the occipital lobe (between the cuneus and the frontal lobe), and the frontal lobe (between the paracentral lobule and the occipital lobe). Treatment-resistant individuals showed reduced functional connectivity in the temporal lobes as regions most implicated. Decreased connectivity between frontal and temporal brain hubs regions is a particularly vulnerable circuit consistently reported in several studies in schizophrenia and is also characteristic of the circuit pathophysiology of TRS (80).

In terms of network-based analysis, global network efficiency was significantly reduced in the TRS group compared to controls with significant increases in local efficiency. Reduced global network efficiency indicates that the reduction of functional connectivity and integration between different brain hubs in TRS as a result of the disease process may create surrogate or back-up connections locally (increase in local efficiency) as a homeostatic mechanism and an attempt to compensate for the reduction in longer-range connectivity and restore integration (46, 80).

## The synchronization of cortical circuits

One possible functional correlate of the aberrant connectivity observed in TRS is disturbances in cortical network oscillations. Oscillations in network activity include the theta (~4–8 Hz), alpha (~8–13 Hz), and gamma (~30–80 Hz) bands. These oscillations are measurable by electroencephalography (EEG) and magnetoencephalography (MEG) and are thought to be reflective of cortical information processing and integration (79, 81) Importantly, they reflect the synchronous activity of large populations of neurons that integrate information across multiple brain regions. With regard to schizophrenia, specific interest has been paid to the gamma band oscillation (GBO) (82–85). The GBO plays an important role in a variety of cognitive tasks including sensory processing, working memory, attention, and cognitive control–all of which are disturbed in the illness (86–91). More generally, it is thought to be critical to the process of feature binding, in which sensory information of a variety of modalities is integrated coherently into a unified representation (92). Fittingly, it has been suggested that the underlying dysfunction in schizophrenia is the inability to integrate the activity of distributed neuronal networks. These disturbances in the GBO could underlie the dysfunctional communication observed between disparate brain regions in the illness.

The GBO has been shown to be disrupted in schizophrenia patients during the performance of a wide variety of tasks, including simpler perceptual tasks and more complex and cognitively demanding tasks (93–96). In patients diagnosed with schizophrenia, EEG studies have shown that the GBO is impaired in working memory tasks at frontal and posterior sites, as well as in the frontal cortex during cognitive control tasks (97–100). Performance of these tasks is typically associated with increase in GBO activity in healthy subjects. However, in subjects with schizophrenia this demand-related modulation of the GBO is absent or diminished. The deficit in task-related modulation of the GBO is also present in first-episode patients, suggesting that this is driven by the underlying disease process rather than illness chronicity or long-term use of antipsychotic medications (99). Several of these studies have also shown that deficits in cognitive control in patients with schizophrenia are correlated with their deficits in GBO activity (91, 98). Convergent evidence from fMRI studies has also shown a lack of task-demand related modulation of activity in the PFC in schizophrenia patients (101). These findings suggest that for cognitive tasks, particularly those that may depend on integration of information, the GBO is a reflection of disturbed functional connectivity between communicating brain regions.

Multiple models have been generated to describe the underlying neural circuitry that gives rise to the GBO. Two prominent ones include the Interneuron Network Gamma (ING) model and the Pyramidal Interneuron Network Gamma (PING) model (102). In the ING, pyramidal cells are synchronized by the activity of interneurons, but pyramidal cells themselves are not directly involved in the generation of the GBO. In PING, oscillations are generated via the recurrent synaptic connectivity between the excitatory activity of pyramidal cells and feedback inhibition of interneurons. While this process is still not fully understood, experimental observations favor the PING model of GBO generation. In this case, synaptic inhibition via GABAergic interneurons defines the timing and firing rate of pyramidal neurons, creating precise windows within which large groups of excitatory cells can fire synchronously (103–105). In turn, excitatory cells also provide input onto GABAergic interneurons, creating a loop for entrainment of cortical networks across brain regions. Support for the PING model comes from findings that interneuron activity follows pyramidal cell activity by a short delay, consistent with pyramidal cell excitatory drive as the main stimulus for interneuron excitation in the model (106, 107). Within excitatory cells, α1-containing GABA_A_ receptors post-synaptic to a subset of inhibitory interneuron processes produce currents with decay periods fitting for the production of gamma oscillations (84). Lastly, it has been shown that with genetic knockout of AMPA glutamate receptors within specific populations of inhibitory interneurons, synaptic excitation of these inhibitory interneurons is diminished and the power of the gamma oscillation severely reduced (108). These findings support the theory that the GBO arises from a complicated interplay between excitatory pyramidal cells and inhibitory interneurons.

Consistent with the PING model, there is ample evidence to suggest that both excitatory glutamatergic and inhibitory GABAergic activity are disturbed in schizophrenia (84, 85). Deficits in excitatory glutamatergic signaling have been identified as a possible core feature behind the pathophysiology of schizophrenia that gave rise to the NMDA receptor hypofunction hypothesis (71). This hypothesis arose from the observation that NMDA receptor antagonists (e.g., ketamine, PCP) can reproduce some of the symptoms of schizophrenia. Subsequent studies have identified widespread dysfunction of NMDA receptors in schizophrenia. Interestingly, given that the GBO is thought to be generated by the activity of inhibitory interneurons, much of the observed dysfunction in NMDA receptors has been specific to inhibitory interneurons themselves. For example, post-mortem analysis of the PFC of schizophrenia patients has shown a 50% reduction in the expression of the NR2A subunit within inhibitory interneurons that express parvalbumin, a calcium binding protein (109). Moreover, chronic NMDA receptor antagonist administration in rodent models reduces the expression of the parvalbumin protein and GAD67 (the primary GABA-synthesizing enzyme glutamic acid decarboxylase) in parvalbumin-positive(+) inhibitory interneurons (110, 111). Acute administration of NMDA receptor antagonists has also been shown to decrease the activity of interneurons with a corresponding increase in the activity of pyramidal cells (70). Thus, NMDA receptor antagonism may reduce the function of inhibitory interneurons which subsequently disinhibits the activity of pyramidal cells. Within the context of schizophrenia, NMDA receptor hypofunction may result in the diminished excitation of inhibitory interneurons within cortical networks.

**Figure 2:**
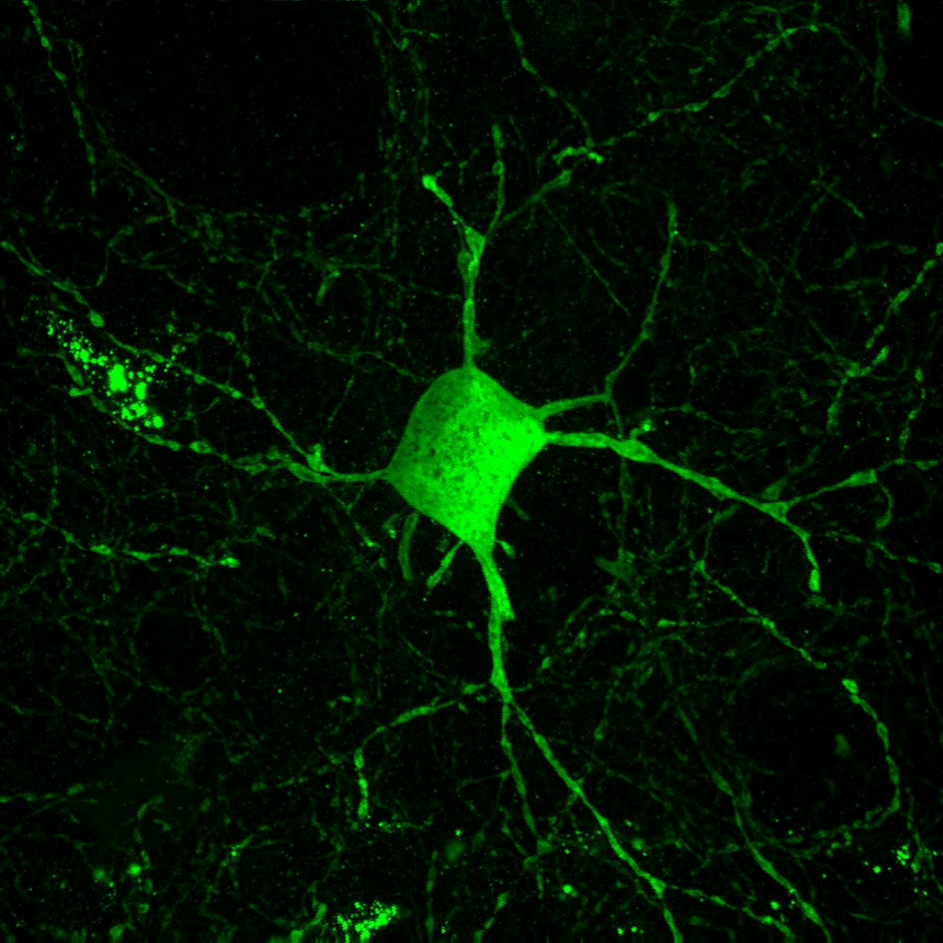
Parvalbumin interneurons contribute to the inhibitory dysfunction in schizophrenia. Parvalbumin interneurons are fast-spiking inhibitory interneurons characterized by the calcium binding protein parvalbumin. These interneurons are innervated by excitatory glutamatergic cells and in turn their projections target the cell soma of excitatory pyramidal cells. This excitatory-inhibitory interplay is thought to give rise to the GBO, which is reflective of parvalbumin interneurons role in synchronizing large populations of excitatory cells. The GBO is disturbed in schizophrenia, and dysfunction within parvalbumin interneurons is thought to be central to these abnormalities.

**Figure 3:**
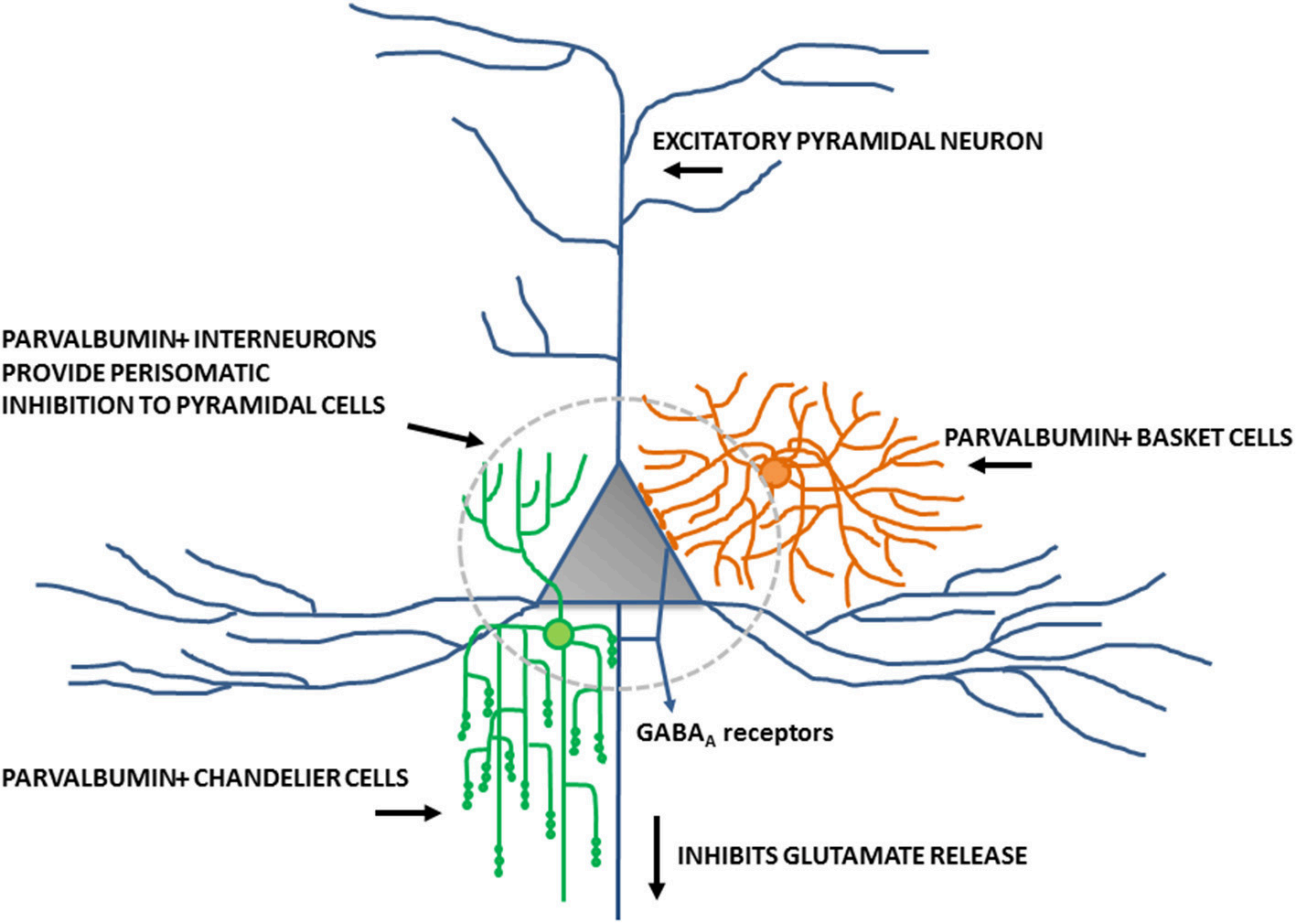
Schematic diagram of perisomatic inhibition of cortical pyramidal cells by parvalbumin+ basket cells and chandelier interneurons. Adapted from Lewis et al. (115).

Inhibitory interneurons are particularly sensitive to NMDA receptor antagonists (70, 112, 113). In combination with findings of altered expression of NMDA receptors within these interneurons, it is well-supported that inhibitory interneurons, particularly those expressing the calcium-binding protein parvalbumin, are a locus for dysfunction in schizophrenia (shown in Figure 2) (84, 114, 115). A number of studies have shown that parvalbumin+ cells are critical to the generation and maintenance of the GBO (106, 113, 116, 117). These interneurons have extremely fast-spiking properties and their rapid synaptic activation is consistent with the frequency required for entrainment of the GBO (118). Parvalbumin+ cells also show the strongest coupling to the gamma oscillation cycle relative to other interneuron types (e.g., calbindin, calrentin) (119, 120). Parvalbumin+ interneurons are typically fast-spiking and provide perisomatic inhibition onto excitatory pyramidal cells. Parvalbumin+ interneurons can present morphologically as either basket (project to the soma and proximal dendrites of neurons) or chandelier cells (project to the initial axon segment of neurons) as illustrated in Figure 3. While both parvalbumin+ basket and chandelier cells are active during GBO, parvalbumin+ basket cell activity is more strongly coupled with the GBO (121). Studies have also shown that GBO power is markedly reduced by opioid receptor activation, which dampens the activity of synaptic inputs from parvalbumin basket cells onto pyramidal neurons but does not affect chandelier neurons (122). These findings emphasize the critical importance of parvalbumin+ basket cells specifically to the generation of the GBO and their dysfunction in schizophrenia. In support of this, it has been shown that reductions in the firing rate of parvalbumin+ interneurons via optogenetics can reduce the power of GBO (114). Conversely, non-rhythmic stimulation provided to parvalbumin+ interneurons can increase the power of the GBO.

Parvalbumin+ cells have been extensively studied in schizophrenia and evidence of their dysfunction extends well beyond their contribution to the GBO (85, 115, 123). Parvalbumin+ cells have a reduction in mRNA and protein levels of parvalbumin itself despite unaltered neuronal density in patients with schizophrenia observed post-mortem (124–126). Parvalbumin+ cells also have reduced protein and mRNA levels of GAD67 and up to 50% of parvalbumin+ cells are wholly devoid of GAD67 (124). This loss of GAD67 represents a significant decrease in the strength of inhibitory inputs on the pyramidal cells they target (115). Moreover, this deficit has been observed in parvalbumin+ cells across multiple cortical regions including the DLPFC and ACC (127–129). Two hypotheses have been generated to account for the convergent evidence of dysfunction localized to parvalbumin+ basket cells (84). One hypothesis emphasizes the inhibitory contribution to this network interplay and the other excitatory activity. First, lower GAD67 levels in parvalbumin+ basket cells could result in a disinhibition of pyramidal cells. Alternatively, the loss of GAD67 in parvalbumin+ basket cells could be a development disruption due to lack of excitatory input onto these cells to drive their activity. Consistent findings of dendritic spine loss on pyramidal cells in areas like the DLPFC and dysfunction within glutamatergic channels (e.g., NMDA, AMPA) could contribute to this loss of excitatory input onto parvalbumin+ basket cells in schizophrenia (68). These findings support the central importance of parvalbumin+ inhibitory interneurons in schizophrenia pathophysiology but whether this is a primary pathology or homeostatic mechanism in response to diminished pyramidal cell input is still unclear.

Despite an improved understanding of the underlying pathophysiology of schizophrenia, particularly with regard to cellular mechanisms contributing to the GBO, a multitude of questions remain. Of utmost importance to the current review is the validity of these findings, many of which have been garnered from animal models of schizophrenia, to TRS. Current cellular and animal models have significant limitations in modeling the illness and few, if any, attempts have been made to replicate the treatment-resistant presentation of the disorder. Secondly, further investigation is required to understand the complex interplay between excitatory glutamatergic cells and inhibitory interneurons in the dysfunctional circuitry of schizophrenia. Specifically, a better understanding of the cellular properties that give rise to the GBO are necessary to better understand approaches for treatment. And lastly, novel treatments and therapeutics need to be designed to target the pathophysiological functioning of GBO circuitry. These approaches may include pharmacological stimulation of the neural circuitry or might be targeted through novel non-pharmacological approaches, such as rTMS which can directly stimulate the GBO.

## Symptom domain circuits

Patients who have a phenotype of psychosis that is responsive to dopamine-blocking medication may have dysregulated striatal hyperdopaminergia related to circuit abnormalities within the fronto-striatal complex of the mesolimbic dopaminergic pathway. Glutamatergic projections from the PFC to the ventral tegmental area (VTA) normally regulate dopamine release in the nucleus accumbens. Within this circuit phenotype, hypofunctioning NMDA glutamate receptors on cortical parvalbumin+ GABAergic interneurons will cause an excessive release of glutamate within the VTA. Hyperglutamatergia then leads to overstimulation (on circuit phenotype) of dopaminergic neurons within the mesolimbic dopamine pathway and excessive release of dopamine within limbic structures, such as the nucleus accumbens, amygdala and hippocampus (130, 131). Hyperdopaminergia within the fronto-striatal circuit underlies the beneficial positive symptom domain response that treatment-responsive patients achieve with D_2_-blocking medications.

Negative and cognitive symptom domain circuitry involves cortical brainstem glutamate projections that communicate within the mesocortical dopamine circuit. Glutamatergic projections from the cortex onto hypofunctioning NMDA glutamate receptors located on cortical parvalbumin+ interneurons leads to the excessive release of glutamate in the VTA. The excessive stimulation of pyramidal VTA neurons then leads to the inhibition (off circuit phenotype) of mesocortical dopamine neurons and insufficient dopamine release in the PFC and subsequent negative and cognitive symptoms in schizophrenia (130, 131).

In those patients who fail to respond to antipsychotic medication, it has been demonstrated that although D_2_ receptor occupancy is identical to treatment-responsive patients, the lack of efficacy from D_2_-blocking medication may indicate that hyperdopaminergia may not be related to the symptoms associated with non-response to medications (132). Higher levels of striatal dopamine synthesis capacity have been found in patients with schizophrenia who responded to treatment vs. those patients with TRS who have much lower striatal dopamine levels comparable to healthy controls (72). Also, fronto-striatal dysconnectivity is more pervasive and widely distributed anatomically in TRS as compared to treatment-responsive individuals which may also explain the limited efficacy of dopamine-blocking medication targeting D_2_ receptors within the fronto-striatal circuit in TRS (133).

The neurobiology unique to treatment resistance may involve more glutamatergic related abnormalities than disruptions involving dopamine. Clozapine has a unique and complex pharmacological profile (having a higher affinity to D_4_ receptors than to D_2_ receptors) and a higher binding affinity to many other non-dopaminergic receptors. Clozapine is able to normalize glutamate neurotransmission by increasing NMDA receptor activity in the cortex by a number of different mechanisms. It has been demonstrated that antagonism of D_4_ receptors can regulate glutamatergic transmission by upregulating AMPA receptors and providing homeostatic stabilization of the excitation of PFC pyramidal neurons by indirect enhancement of NMDA activity (134). Clozapine has also been shown to reduce the reuptake of glutamate in the cortex by decreasing the expression of glutamate transporters located on both glial and neuronal cells in cortical and subcortical areas (135). Clozapine has the ability to antagonize glycine transporter-1 (GlyT1) sites for reuptake of glycine by glial cells (136), and can increase glial D-serine release and enhance the release of glutamate via activation of NMDA receptors (137) which may help to regulate some of the downstream glutamate abnormalities that have been found in TRS (73, 74).

It is difficult to map the underlying circuit pathology in ultra-resistant schizophrenia due to the heterogeneity of the illness and limited studies that have explicitly examined this population. Due to multidimensional symptom domains resistant to clozapine, ultra-resistant schizophrenia can be described as the most severe phenotype of the illness that is mediated by multiple mechanisms far beyond dysregulated striatal hyperdopaminergia and glutamate NMDA receptor hypofunction.

## Circuit-based pharmacological treatments

Currently, there are no customized neural circuit-specific and targeted therapies that can address the neural-dysconnectivity in schizophrenia. Despite the lack of precision and ubiquitous targets of pharmacological methods, the use of adjunctive agents to antipsychotic medications may be conceptualized within a circuit context to help improve neuronal network integration and treatment response in TRS. In many cases, augmentation strategies are needed to improve the residual psychopathology symptom domains that have been non-responsive to antipsychotic drugs (including clozapine). Usually in those patients who have not responded to clozapine, a variety of other antipsychotic medications, antidepressants, anticonvulsants, benzodiazepines or a variety of glutamate augmenting agents have been attempted. Clinical studies have used a variety of agents that can enhance glutamate NMDA receptors (on connectomic) function in an attempt to improve downstream GABAergic (off connectomic) inhibitory effects. GABA interneuron modulators have also been recently investigated as an attempt to inhibit pyramidal cell firing, as well as NO-based therapies to improve intracellular NMDA receptor signaling and other direct circuit-targeted neurosurgical and neuromodulation strategies for their therapeutic benefit in treatment resistant disease.

## Glutamatergic agents

Many drugs that target and co-activate glutamatergic pathways have been of interest as a non-dopaminergic approach to improve antipsychotic treatment in schizophrenia. Strategies to improve glutamate NMDA receptor hypoactivity on GABAergic interneurons have targeted extracellular binding sites on the receptor. The glycine modulatory site has been investigated as a target to improve NMDA receptor hypofunction in schizophrenia and several agonists or partial agonists of this binding site on the NMDA receptor have been studied in clinical trials (138).

The amino acid glycine is a co-agonist of the NMDA receptor and it is required along with glutamate to activate the NMDA ion channel (139, 140). The binding site for glycine (located on the NR1 subunit) of the NMDA receptor was first discovered by Johnson and Ascher (1987) by preclinical electrophysiology studies using the outside-out patch clamp method. The NMDA receptor response was then observed to be potentiated by glycine. The distinct binding site (glycine B receptor) was separate from the strychnine-sensitive glycine inhibitory receptor as NMDA receptor potentiation by glycine was not blocked by strychnine (139). In clinical studies, reduced plasma concentrations of glycine have been found in patients with schizophrenia and have been correlated with a greater number of negative symptoms (141, 142), supporting the use of glycine as a strategy to improve NMDA receptor functioning in those patients identified as having treatment resistance specific to the negative symptom domain (138).

Glycine was first used as an augmenting treatment in schizophrenia close to 30 years ago in a few small open-label clinical trials used at doses between 5 and 25 g per day (138, 143–145). In subsequent controlled trials, 60 g of glycine augmented with first-generation or second-generation antipsychotic medication was reported to improve not only the negative symptoms (146–150), but also cognitive symptoms (147, 148, 150) and the depressive symptoms of the illness (148). Glycine is not able to cross the blood-brain barrier easily as it has no specific amino acid transporter, so higher doses must be used that impacts patients' tolerability to glycine. The benefits reported of using glycine as an augmenting treatment to antipsychotic medications to improve the cognitive and negative symptoms domains of the illness has since been disputed. In a subsequent review, glycine was found to have moderate effect in reducing negative symptoms and it was uncertain whether it had any benefit at improving cognitive symptoms (151). The multicentre Cognitive and Negative Symptoms in Schizophrenia Trial (CONSIST), found no significant differences between glycine and placebo at improving the negative or cognitive symptom domains of the illness (152). Overall, glycine may be beneficial for those patients that have treatment resistance specific to the negative and cognitive symptom domains; (153) however it has not been a beneficial augmenting strategy in patients with TRS on clozapine (154).

An alternative approach to increasing endogenous brain glycine concentrations has been to block its reuptake and thus improve glutamatergic tone. The amino acid sarcosine, a GlyT1 inhibitor, has also been demonstrated to improve the negative, cognitive and depressive symptom domains of schizophrenia (155, 156). Unfortunately, significant side-effects have since been reported including ataxia, hypoactivity and respiratory depression with the use of sarcosine, perhaps in relation to mechanisms involved in the overstimulation of the strychnine-sensitive glycine inhibitory glycine receptor (157, 158). When used as an augmenting strategy in patients with TRS, sarcosine was also not effective (159). This may be related to clozapine's glutamatergic effects and known GlyT1 antagonist properties (136, 138). Bitopertin, a non-sarcosine-based selective GlyT1 inhibiting drug, has also been investigated as an adjunct to antipsychotics (at doses of 10 and 30 mg per day) to mainly target the negative symptom domain of the illness (160). In subsequent phase III trials (SearchLyte trial programme), bitopertin was unsuccessful at improving the primary outcome measure of Positive and Negative Syndrome Scale (PANSS) (161) negative symptom scores over placebo which led the manufacturer Hoffmann-La Roche to discontinue the programme prematurely (138).

D-serine, an allosteric modulator at the glycine co-agonist binding site, has also been investigated as an augmenting strategy primarily for improving the deficit symptoms of schizophrenia. D-serine may be more effective than glycine as it has a greater affinity for the glycine/serine binding site and also has an increased ability to cross the blood-brain barrier (162–164). Serum concentrations of D-serine have also been found to be reduced in schizophrenia (165). D-serine selectively binds to synaptic NMDA receptors and may strengthen circuit connectivity and have more of a neuroprotective effect as compared to glycine, which binds to both synaptic and extrasynaptic NMDA receptors (138, 166). The therapeutic effects of D-serine to improve refractory negative symptoms in schizophrenia have been demonstrated when added to antipsychotic therapy in patients with acute (156), chronic (167), and treatment-resistant illness (168). D-serine is well-tolerated and has been reported to be safe and effective used at dosages up to 120 mg/kg per day (169). D-cycloserine, a drug that was initially used to treat tuberculosis and an anolog of D-serine, is also active at the glycine site and has been reported to benefit the negative symptom domain of schizophrenia (170–172). Unfortunately, in patients with TRS, glycine, D-serine, and D-cycloserine have all been reported to be less effective at improving the negative and cognitive symptom domains in those patients receiving clozapine therapy (138, 152, 154, 172, 173).

Drugs that can downregulate presynaptic disinhibited glutamate release on secondary downstream glutamate neurons have also been explored in patients with TRS and may also work to modulate circuit connectivity. Lamotrigine, an anticonvulsant drug that suppresses presynaptic glutamate release by the blockade of voltage-sensitive sodium channels has been shown to improve clinical response when used as an adjunct to clozapine treatment in ultra-resistant schizophrenia (138, 174–178). The beneficial effects may be associated with clozapine's low affinity to the D2-receptor and involvement with the glutamate system (in comparison to other antipsychotic drugs) which may be further enhanced by lamotrigine (138, 175). More recent clinical trials have studied the efficacy between the metabotropic glutamate 2/3 (mGlu2/3) receptor agonist pomaglumetad methionil (also known as LY2140023) and atypical antipsychotics (138, 179, 180). In a phase II study, it was found to be less effective than the comparator atypical antipsychotic (180) and Eli Lilly subsequently stopped a phase III trial investigating the compound as it failed to meet its primary endpoint.

## Nitric oxide-based treatments

An alternative and novel approach that may improve glutamate NMDA receptor signaling and circuit connectivity in schizophrenia is to target the glutamate-NO-cyclic guanosine monophosphate (cGMP) signaling cascade. Nitric oxide is produced in the brain by a complex interaction with a functional glutamate NMDA receptor and there have been a number of clinical studies suggesting that signaling within the glutamate-NO-cGMP pathway may be disrupted in the illness (138, 181–187). As a gaseous signaling molecule, NO is classified as a neuromodulator or second messenger due to its ability to generate the production of cGMP. Nitric oxide-mediated signal transduction is an important driver for a variety of cellular processes throughout the body, including those critical for the establishment and maintenance of functional neuronal circuits and synaptogenesis (138, 188). In the cerebral cortex, neurons that produce NO are among the earliest differentiating cells that develop (138, 189). The presence of NO-producing neurons during critical developmental growth periods suggests that NO may be required for the formation and subsequent migration of neurons in the brain, and interruption of NO synthesis could lead to impairment in neuronal connectivity as is observed in schizophrenia.

Studies examining the effects of the NO donor drug sodium nitroprusside (SNP) in PCP-treated rats has contributed insight into the role of NO in psychosis (138, 190, 191). The results then stimulated the investigation of the therapeutic effects of SNP in schizophrenia (192, 193). Sodium nitroprusside is a nitrovasodilator drug traditionally used for hypertensive crisis (194). When SNP is administered, it reacts with oxyhemoglobin molecules that are within erythrocytes to form methemoglobin which causes the molecule to become unstable and immediately release NO (138, 194).

The first investigational clinical trial of NO in schizophrenia was conducted at the University Teaching Hospital in Ribeirao Preto, Sao Paulo, Brazil. In this clinical trial, an intravenous infusion of SNP in patients who were already on antipsychotics produced rapid improvement of symptoms (within 4 h of a single infusion) as compared to those patients who received a placebo infusion (138, 192). Symptom improvement continued for 4 weeks following the infusion (although antipsychotic medication adjustments were permitted 7 days following the infusion). The lasting benefits are thought to be related to cGMP's ability to stimulate early gene products and subsequent modulatory effects on the NMDA receptor itself. Sodium nitroprusside has been beneficial in both early stage schizophrenia and in a few case reports of ultra-resistant schizophrenia and did improve a wide spectrum of symptom domains, including the positive, negative, and anxiety symptoms of the illness (138, 192, 193). The results were not replicated in a subsequent trial testing SNP in a population of long-term chronically ill patients (195), which may suggest that SNP-based therapies may be most effective when used within the earlier stages of the illness in those patients experiencing acute symptoms.

In relation to these findings, Dr. Paul Morrison (King's College London) is currently testing the NO-based compound glyceryl trinitrate (GTN) for its ability to improve the cognitive symptom domain of patients experiencing acute psychosis and who are requiring hospitalization (Clinicaltrials.gov Identifier: NCT02906553). Glyceryl trinitrate is another nitrovasodilator drug that has been used to treat angina and other cardiac conditions including myocardial infarction and congestive heart failure. The biotransformation of GTN involves both enzymatic and nonenzymatic pathways that are linked to the pharmacokinetic and pharmacodynamics properties of the drug (138, 196). The metabolic conversion of GTN to NO may also improve downstream glutamate signaling. This clinical trial aims to assess the role of the NO system in cognition and will initiate a sublingual GTN spray 0.4 mg dose, once per day for 3 days or matching placebo formulation spray not containing GTN before the patients are initiated on antipsychotic medication. Glyceryl trinitrate in sublingual spray formulation is a much more convenient and less invasive approach to drug delivery than intravenous infusion of SNP in patients with schizophrenia and may be a promising approach to further improve treatment-resistant cognitive symptoms in the illness.

## GABA_ergic_ interneuron modulators

Pharmacological strategies that target GABAergic interneurons that may correct dysfunctional inhibitory feedback within corticolimbic circuits are also being investigated. Specifically, parvalbumin+ cells are now also being explored as a novel approach to repairing DLPFC neural circuitry and improving the cognitive symptom domain in schizophrenia (Clinicaltrials.gov Identifier: NCT03164876). Parvalbumin+ cells innervate multiple pyramidal cells and contain lower mRNA for parvalbumin and GAD67 in those with schizophrenia (124) and reduced expression of the potassium channel *KCNS3* gene which encodes the Kv9.3 potassium channel α subunit and is essential for control over its fast-spiking abilities (197). Inhibitory parvalbumin+ interneurons contribute to the cognitive deficits in schizophrenia (115) and in unmedicated patients with the illness. Kv3.1 channels located on parvalbumin+ cells are reduced by disease and then normalized with the use of antipsychotic drugs (198). Dr. Charles Large (Autifony Therapeutics) has recently completed a phase I study of AUT00206, a Kv3.1 channel modulator in healthy volunteers (Clinicaltrials.gov Identifer: NCT02589262) and in collaboration with Dr. Oliver Howes (King's College London), his team are currently recruiting for a continued phase I study to explore its safety, tolerability, pharmacokinetics and treatment effects on relevant biomarkers in patients with schizophrenia (Clinicaltrials.gov Identifier: NCT03164876).

## Circuit-based neurosurgery

Surgical modalities that can precisely target particular regions of focal and well-localized dysconnectivity in the brain are currently being tested as a more circuit-specific approach to precision medicine in schizophrenia. Deep brain stimulation (DBS) has been a well-established targeted therapeutic approach that has been used to improve the treatment-resistant symptoms of Parkinson's disease, obsessive-compulsive disorder and treatment refractory depression (199–202).

Neurosurgical DBS strategies are also now being considered to be used in ultra-resistant schizophrenia to target those relevant brain hubs that may improve the interconnectivity of relevant neuronal circuits. The implantation of electrodes into accessible anatomical nodes can be targeted to normalize or reset abnormal patterns of cortical network GBO activity that disrupt neural circuits. The stimulation settings of the electrodes can be titrated to tune the neurons to specific frequencies and recalibrate neuronal asynchrony. There is current interest in targeting several important network hubs using DBS in ultra-resistant schizophrenia involved in basal ganglia-thalamocortical and DLPFC brain circuits. Hubs identified include the hippocampus, ventral and associated striatum, medial and DLPFC, substantia nigra, nucleus accumbens and the mediodorsal nucleus of the thalamus (203–205). These hubs have been chosen primarily based on known pathological findings in schizophrenia and/or their interconnectedness to other brain hubs that are circuit-specific and related to the excessive and mistimed dopamine release in the striatum. Hippocampal dysfunction that drives downstream dopamine release in the striatum contributing to persistent positive symptoms is one of the clinical hallmarks for treatment-resistant disease (206).

Currently there are two phase I DBS trials investigating this approach in ultra-resistant schizophrenia that are recruiting patients. The first trial at Hospital Santa Creu i Sant Pau in Barcelona (Clinicaltrials.gov Identifier: NCT02377505) is targeting electrode placement in either the nucleus accumbens or the subgenual ACC. The participants will be randomized to receive stimulation to either of these neuroanatomical sites with the stimulation remaining on until a full 6 months of stabilization is achieved. Those patients who are responsive will then be crossed-over to stimulation-on or stimulation-off groups for 3 months.

The principal investigator, Dr. Iluminada Corripio has recently reported positive findings in the first subject who participated in this clinical trial. The patient had a long history of ultra-resistant schizophrenia-positive symptom domain refractory symptoms including manifestations of persecutory, control and delusions of reference. Her referential delusions had become so pronounced that she was unable to leave her home. The patient had a long treatment history typical of ultra-resistant schizophrenia including many trials with a number of different antipsychotic medications, including the use of clozapine (600 mg/day) with little benefit. The patient underwent bilateral electrode implantation in the nucleus accumbens and left-sided unilateral stimulation. Improvement was achieved in both positive and negative symptoms measured 4 weeks post-implantation and after 11 months of open treatment, the patient experienced over a 60% reduction in positive symptoms as measured by the positive symptoms subscale of the PANSS as well as a 33% reduction in negative symptoms, 50% reduction in the PANSS disorganization factor, 33% reduction in PANSS excited factor and 16.7% increase in the depressed factor. The patient continues to do well and is now able to leave her home and has made significant improvements to her overall functioning. For this patient with ultra-resistant schizophrenia, this DBS treatment option was of substantial benefit to otherwise untreatable refractory symptoms (207).

The second DBS trial in ultra-resistant schizophrenia is out of Johns Hopkins University where the study team led by Dr. William Anderson will be recruiting three ultra-refractory patients and will be targeting the local inhibition of the substantia nigra pars reticulata (SNr), a major outflow nucleus of the basal ganglia with the intention of disinhibition and driving the activity of the mediodorsal nucleus of the thalamus (Clinicaltrials.gov Identifier: NCT02361554). The structure and hypofunction of the mediodorsal nucleus of the thalamus has been investigated in several imaging and post-mortem studies in schizophrenia (208). All of the DBS studies in ultra-resistant schizophrenia are only recruiting those patients who have exhausted all other therapeutic alternatives and continue to have severe and disabling clinical symptoms and poor functioning.

## Circuit-based neuromodulation

The use of external neuromodulation devices, a less invasive circuit-based treatment approach than DBS has also become an alternative treatment option for refractory schizophrenia. Repetitive transcranial magnetic stimulation (rTMS) has been the method most investigated. In rTMS time-varying currents are generated in an induction coil and are held over the scalp and applied to stimulate and improve the functioning and synchrony of the GBO networks and GABA inhibitory mechanisms within the brain circuits beneath it. There have been several randomized studies conducted to show that stimulation targeted over the left TPJ, a critical hub involved in the pathophysiology of AVH, can reduce these symptoms (209–215).

Transcranial direct current stimulation (tDCS) is an alternative non-invasive form of neuromodulation that has been used to target specific circuits of the brain to improve treatment-refractory symptom domains of schizophrenia. It is a smaller, lightweight, portable and less expensive option than TMS and could be easily used at home to reduce the burden of having to receive daily treatments within a clinical setting (216). In this approach, two sponge electrodes are positioned on the scalp to facilitate a low-intensity electrical current (1–2 mA) that is passed between them. The transcranial current that is generated is continuous and flows in a direct current from an anode (current that enters the body) to induce prolonged depolarization to a cathode (a current that exits the body) to induce hyperpolarization under the cathode (217–220). It is thought that the mechanisms involved in the longer-lasting effects of tDCS are protein synthesis-dependent and in the modification of intracellular cascades beyond the membrane potential to influence cellular features associated with NMDA receptor functioning (216, 217). tDCS is increasingly being investigated by more independent schizophrenia researchers and primarily for improvement of positive (AVH) and negative symptom domain refractory symptoms.

Based on observations of the dysconnectivity of fronto-temporal circuits from functional neuroimaging studies of patients experiencing AVH (60–62), clinical studies have used tDCS to improve the dysconnectivity of these circuits to decrease AVH in patients with schizophrenia. In these studies, the anode electrode is applied over the left DLPFC (abnormally hypoactive) with the cathode electrode applied over the TPJ (abnormally hyperactive) to modulate the circuit and alleviate the severity of the AVH in schizophrenia (218, 221, 222). Results have been mixed in the ability of tDCS to reduce severity and frequency of AVH. For reviews see Li et al. (223), Ponde et al. (224), and Agarwal et al. (225). Studies that have reported a stronger and longer lasting response have had a higher number of treatment sessions and/or shorter time interval between sessions within their design (221, 226).

Open-label and randomized clinical trials that have examined the effects of tDCS to target negative symptoms of schizophrenia have placed the anode over the left DLPFC and the cathode over the right DLPFC or the right supraorbital region or placed it extra-cephalically (221, 227–229). A meta-analysis concluded that tDCS treatment is beneficial for improving negative symptom domain indications (211). There has been direct support for the safety of tDCS in human clinical trials with the most often reported side-effect of mild skin erythema, itching, tingling and burning under the electrode placement as well as temporary headache and dizziness which resolves after stimulation (218, 220).

## Concluding remarks

Treatment resistance in schizophrenia continues to be a therapeutic challenge in psychiatry. Within the spectrum of the disease, neural circuits within specific brain regions and their structural and functional links to corresponding regions seem to be further disrupted in TRS. In this review, we have examined TRS from a circuit-based perspective. We highlighted attempts by leading schizophrenia clinicians and researchers to standardize the definition of treatment resistance in schizophrenia and have identified and incorporated recommended terminology with regards to the clinical sub-specifiers or symptom phenotypes that are common to TRS. We discussed the developments of network-based science from the early pioneers who recognized psychiatric illness and schizophrenia as a disease of neuronal and functional disconnectivity. With the development of neuroimaging methods, modern-day connectionists have built upon these theories and have continued to develop and advance network connectomic science today.

Our review of schizophrenia and TRS within a connectome context suggests that the structural and functional alterations may be greater in those patients with persistent treatment-resistant symptoms, indicating that there may be fundamental differences within brain network properties that contribute to the inability to integrate the activity and function of distributed neuronal networks that are specific to TRS. Cortical network oscillations and GBO in particular have been reviewed to understand their role in the integration of neuronal information across large neuronal ensembles in the illness. The complex relationship involved in the synchronized firing between excitatory pyramidal cells and inhibitory GABAergic interneurons were also reviewed, including findings specific to dysfunctional inhibitory networks in schizophrenia and parvalbumin interneuron dysfunction and what role these cells may play in dysfunctional pyramidal cell inhibition in schizophrenia.

We conclude the review with an overview of several augmenting pharmacological treatments, such as glutamate NMDA receptor and GABA interneuron modulators as well as NO-based treatments and how they may be viewed within a circuit context. Neurosurgical and neuromodulatory approaches were also discussed to highlight a number of beneficial circuit-based targets that may improve circuit integration and treatment response in TRS and improve treatment refractory symptoms in patients who have demonstrated poor response to alternative treatment approaches. The precise mapping of cellular and system-level networks to both on (excitatory) and off (inhibitory) circuit phenotypes specific to treatment-resistant disease remains challenging. Understanding the complexity of the cellular properties that are involved in dysfunctional brain networks in TRS will be critical toward future research in neural circuit-specific pharmacotherapeutics and directed neuromodulation treatments in schizophrenia. The ongoing interest and innovation that has been dedicated toward the understanding of the neural circuitry of schizophrenia and targeted treatment of TRS will hopefully improve personalized outcomes of those suffering from this debilitating disease.

## Author contributions

M-AM, JP, and JW conducted the literature review. M-AM and JP wrote the first draft of the review. JW, IW, GB, and SD all contributed to and approved the final manuscript.

### Conflict of interest statement

The authors declare that the research was conducted in the absence of any commercial or financial relationships that could be construed as a potential conflict of interest.
